# Medical Worthies of the West Country
*Presidential address to Bristol Medico-Chirurgical Society, 12th October 1955.


**Published:** 1956-07

**Authors:** Percy Phillips


### MEDICAL WORTHIES OF THE WEST COUNTRY*

BY

PERCY PHILLIPS, M.D., CH.B., M.SC.

"As there is no man can tell who first contrived the use of houses and clothes to defend us from the injuries of the weather, so the beginning of the art of physick can be no more discovered than the fountain of the River Nile."

### THOMAS SYDENHAM, 1624–89

So wrote Thomas Sydenham, sometimes called "The English Hippocrates", and the first notable physician we can claim as a west-countryman. He was born in 1624 at Wynford Eagle in Dorset, in the manor house on an estate bought in the reign of Henry VIII for £40. In a family of ten he was the seventh child, cheerful and happy, but soon to know grief and disaster for shortly after commencing his studies at Oxford, war broke out. As his family were Puritans and the Royalists were preparing to occupy Oxford he returned to Dorset. His father was a captain on the Parliamentary side and four brothers held commissions also. Although he himself was only eighteen he probably served in local engagements. His elder brother Francis was killed in action and even his mother was deliberately shot in a minor action near their village.

It was not until 1646 when he was twenty-two years old that Thomas was able to return to Oxford. There he met with a Dr. Thomas Coxe, whom he describes as the promoter and protector of his early studies. Lectures in those days were on the works of Galen and Hippocrates and from the latter Sydenham learned the value of accurate bedside observation. After less than two years at Wadham College he took his medical degree so that his actual qualifications must have been meagre. He became a Fellow of All Souls and, until his marriage in 1655, was Bursar of the college.

Probably his studies continued during this time but there came another interruption in 1650–51 when Charles II landed in Scotland and Commonwealth forces marched to meet him. In this second war Sydenham served not as a doctor but as a captain of cavalry. Now aged thirty-one, married, with no career, he gave up his fellowship and went to London. For his services he got a grant of £600 but made little headway. Then came the Restoration. He retired to Montpellier for six months' study and finally got a licence to practise in 1663.

How did he differ from his contemporaries? He was devoted to his profession, not so much as a career but as a means of serving mankind. Contrast this with a parody current at the time—" The physicians of the present day are like Balaam's ass; they will not speak until they see an angel." (An "angel" being a ten-shilling piece of that period.)

It was not only his attitude to his profession that was unusual, but also his outlook on disease. He regarded diseases as entities that could be differentiated and classified like plants or animals. He turned medical attention to the scientific study of disease and wrote medical monographs which set the pattern for all time.

There was one disease—gout—on which Sydenham had pretty well the last word. He was a great sufferer himself but it became a blessing in disguise and gave him an opportunity to study it in all its phases so that his treatise on the subject remains the classical description of that disease.

* Presidential address to Bristol Medico-Chirurgical Society, 12th October 1955.

He died in 1689 at the age of sixty-five. His tomb in St. James's, Piccadilly, was practically destroyed in the recent war but the broken stones are being reassembled to remain an inspiration for the future.

### THOMAS DOVER, 1662–1742

I would speak next of this pupil of Dr. Sydenham. His connexion was with Bristol, the city which occupies a unique place in the history of the English Poor Law Administration, and Dr. Dover, who will ever be remembered for his famous remedy "Dover's Powder", was the first medical officer of the newly created Bristol Board of Guardians of which I shall have more to say later.

He spent much of his life at sea and I suppose his most famous voyage was to Juan Fernandez, during which he was second captain to Woodes Rogers in the *Duke*, Captain Courtney being in command of the *Duchess*, and William Dampier the Pilot of the South Seas. They left Bristol on 1st August 1708 and sighted the island, after rounding Cape Horn, on 31st January 1709. Many of the crew were sick with scurvy and when Dover put ashore he saw a fire blaze up in the twilight. Thinking a French man-of-war was in the bay he put back to the ships. Next morning, cleared for action, the two ships entered the bay and found it empty. Dover now went ashore again with seven armed men. He soon returned bringing with him a man clothed in goatskins— Alexander Selkirk of Largs in Scotland who had been left on the island four years before by the *Cinque Ports*, a ship he thought unseaworthy. It was he who had lit the fire on the previous evening. I need hardly tell you that this man was the prototype for Robinson Crusoe in Daniel Defoe's famous book published in 1719. We are told that during his stay he killed 500 goats and lived largely on their flesh with crawfish, turnips and cabbage from the cabbage-trees. "On first coming on board his speech was almost unintelligible from long disuse for he seemed to speak his words by halves."

Dampier had been with Selkirk on the *Cinque Ports* and recommended him so strongly that he was at once appointed mate of the *Duke*. Next day tents were set up on shore for the sick men. Selkirk caught two or three goats each day and the scurvy patients rapidly recovered on a diet of goat's meat, turnip tops and watercress. They sailed on 13th February north along the coasts of Chile and Peru to begin the real business of the expedition—the raiding of the Spanish coast. They captured many ships and finally arrived back in England on 14th October 1711 after a voyage of three years and two months.

£14,000 was spent on fitting out the ships but the net profits were at least £170,000. Dover must have received several thousand pounds as his captain's share besides dividends as an owner. As chief medical officer he received £423. Amongst his treasures were two fine silver candlesticks which he sold to the Town Clerk of Bristol, John Romsey, for £114. Romsey presented them to Bristol Cathedral in 1712 and I believe they are still used on the altar there.

An original copy of his book *The Ancient Physician's Legacy to his Country* can still be seen in the University Medical Library.

Thomas Dover was an interesting character, part doctor, part sea captain, part adventurer and possibly part pirate. He achieved a full and adventurous life. On more than one occasion he sailed from Bristol in charge of a ship on some pioneering expedition. His greatest claim to medical fame must be his powder which is still possibly one of the most effective cures for influenza and even the common cold, which, with all our vaunted knowledge, we doctors have not yet conquered. He died on 20th April 1742, aged eighty years and was buried most probably in the vault of the Tracey family at Stanway, a village about twelve miles north-east of Cheltenham but no memorial now remains.

### THE BRISTOL BOARD OF GUARDIANS

The author of this scheme was a Mr. John Cary, a resident in the city and churchwarden of St. John the Baptist in 1679 and 1680. An act establishing in Bristol the first Board of Guardians was introduced into Parliament in 1696. This authorized the provision of a workhouse and the raising, by a rate from six parishes, of funds to provide maintenance for the destitute poor. The main features of that scheme remained until quite recent times for the Board of Guardians continued until 1st April 1930 when as a result of the Health Act of the previous year such boards were superseded by Public Assistance and more recently Social Welfare services.

The Board consisted of the Mayor, the Aldermen and forty-eight Guardians "to be chosen out of the honestest and discreetest inhabitants of the said City". The first election was held on 12th May 1696 and the Guardians set to work with great energy to carry out the provisions of the Act. They assumed semi-magisterial duties and had strangers and disorderly persons apprehended and committed to "Bridewell" for various periods. The keepers of disorderly houses were punished and the Corporation set up a pair of stocks and a whipping post, whilst chains and bolts to fasten disorderly persons to blocks, were provided in a portion of St. Peter's Hospital known as "purgatory". This building, the old mint in Peter Street, was acquired in 1698 for £500 and survived until the disastrous air raid on 24th November 1940 when this exquisite gabled building was utterly destroyed. It was the headquarters of administration for many years and as the boundaries of Bristol grew so the work increased. The Seal of the Bristol Board of Guardians was a most appropriate one. It consisted of a picture of the dome-shaped straw type of beehive, the motto beneath being "Hyemis memores laborant".

At this point I must say a few words about Southmead Hospital.

### SOUTHMEAD HOSPITAL

By an Act of Parliament in 1897 the Bristol Corporation obtained the transfer of the parishes of Stapleton and Saint George and part of Horfield from the Barton Regis Union and also parts of Bedminster and Keynsham Unions, combining the whole of the extended City into one Poor Law Union. It therefore became necessary on 1st April 1898 for the Barton Regis Guardians to provide a new workhouse for the smaller part of the work that remained to them. They acquired thirteen and a half acres of land at Southmead, Westbury-on-Trym, for the erection of this new building at a total cost of £33,000. The foundation stone was laid by the Dowager Duchess of Beaufort on 18th September 1900 and the building was finally declared open on 18th September 1902 by the Rt. Hon. Sir John Dorrington, Bart., M.P.

A further extension of city boundaries in 1912 ultimately engulfed the new infirmary. Barton Regis ceased to exist as a Board and in taking it over the Bristol Guardians decided on a very progressive policy for those days. They enlarged Southmead at an additional cost of £44,000, building modern wards capable of accommodating about 650 "chronic sick" patients.

These new wards were completed about August 1914 and immediately the Bristol Board offered them as a War Hospital. Thus Southmead became part of the 2nd Southern General Hospital for the reception of war casualties. During this first Great War a new operating theatre was added and during the war years upwards of 37,000 wounded were treated in the hospital. The War Office retained control until October 1920 when the hospital was handed back to the Guardians.

Its first Medical Superintendent under civil control was the late Dr. Robert H. Norgate who died in December 1924. My own connexion with Southmead Hospital began in February 1923 when I was appointed assistant medical officer, ultimately being elected Medical Superintendent in April 1925.

With the passing of the Poor Law régime as a result of the Health Act of 1929, Southmead now passed to the control of the Health Committee of the City of Bristol, being taken over on 1st April 1930. Gradually its scope was increased and in the Second World War it took its full share of the casualties arising from the disastrous air raids from 1940 onwards. However, it is interesting to note that the number of these (635) in no way compares with the many thousands admitted during the war of 1914–18.

Since 1948, Southmead Hospital has been further developed to become a large general hospital with the largest maternity department in the whole of the south-west.

### THOMAS BEDDOES AND THE MEDICAL PNEUMATIC INSTITUTE

Thomas Beddoes, our next character, founded the Medical Pneumatic Institute in Bristol in 1793 in Dowry Square, Hotwells. Here diseases of the lung, such as tuberculosis, were treated by the inhalation of oxygen, hydrogen and carbon dioxide. Beddoes has himself been largely forgotten but his colleague and assistant are both remembered.

James Watt the engineer was the colleague, Humphry Davy, the assistant. In 1800, Davy published the result of his work on nitrous oxide which soon became known as "laughing gas". He wrote "as nitrous oxide in its extensive operation appears capable of destroying physical pain, it may probably be used with advantage in surgical operations."

Unfortunately, little attention was paid to Davy's work. Laughing gas was too funny to be taken seriously!

Literary men of the time, Wordsworth from Alfoxden, near Holford, Southey and Coleridge came to Beddoes' Pneumatic Institute, not to be cured of any disease but to seek the exhiliarating effects of doses of the new gas. Nitrous oxide is still used extensively with oxygen or air as one of the safest anaesthetics. It brings relief to countless thousands in childbirth; it is the only anaesthetic which a midwife is permitted to use on her own responsibility—in a special machine called "Minnitt's apparatus". Had Davy's work and wisdom been followed up, anaesthetics would have been available at least forty-six years earlier, for it was not until 1846 that ether and later chloroform first came into use.

To digress for a moment, James Young Simpson in Edinburgh first used ether successfully on 19th January 1847 in delivering a woman of her second child. In March, he published a paper recording the use of ether in several cases.

Chloroform was discovered in 1831 by Liebig in Germany. It was first used by Simpson in November 1847. Then commenced the battle over chloroform. Dublin contended that it would increase the incidence of bleeding, convulsions, paralysis, pneumonia and so on. Simpson indignantly flourished figures over the heads of his critics showing that shock and exhaustion were much greater dangers than chloroform.

Then the moralists joined the fight and even brought religious objections. Simpson's opponents took their stand on Genesis iii. 16: "Unto the woman he said, I will greatly multiply thy sorrow and thy conception; in sorrow thou shalt bring forth children." Simpson, well versed in the Bible, soon countered this with the first Biblical operation (Genesis ii. 21): "And the Lord God caused a deep sleep to fall upon Adam and he slept; and He took one of his ribs and closed up the flesh instead thereof." Before the clergy had time to work this out, he published a famous pamphlet: *Answer to Religious Objections advanced against the Employment of Anaesthetic Agents in Midwifery and Surgery*.

This was headed by the texts: "For every creature of God is good, and nothing is to be rejected if it be received with thanksgiving." (1 Timothy iv. 4.) "Therefore to him that knoweth to do good and doeth it not, to him it is sin." (James iv. 17.)

Medical, moral and religious objections were swept aside and more and more chloroform was used. The most progressive lady of the time—good Queen Victoria—dealt the final blow, for in April 1853 Prince Leopold was born to Her Majesty whilst she was under the influence of chloroform!

Her physician, Sir James Clark, wrote to Simpson to his great delight: "The Queen had chloroform exhibited to her during her recent confinement. It was not at any time given so strongly as to render the Queen insensible, and an ounce of chloroform was scarcely consumed during the whole time. Her Majesty was greatly pleased with the effect and she certainly never had a better recovery." I like to think that Bristol and the West Country had a share in this romantic battle against pain, and to Thomas Beddoes should go some of the credit for his initiative.

### EDWARD JENNER, 1749–1823

This most famous of all general practitioners is the most illustrious name I can think of associated with the West Country. He lived through the turmoil of the Napoleonic Wars, when the whole of the civilized world was ravaged by a war of almost unequalled ferocity. Before he died, he had the supreme satisfaction of knowing that his discovery had been the means of saving more millions of lives than had been sacrificed during that conflict. Before his time, no one had conquered a disease and most treatments were directed to the relief of symptoms.

The disease he conquered was smallpox. Nowadays the fear of smallpox has been so completely banished that it is difficult to realize what a haunting dread it once was. However, in the *Gloucester Journal* of 23rd February 1823 we read in an obituary notice of Dr. Jenner that 40,000 people died annually of smallpox in the British Isles, that this disease killed one in fourteen of all who were born and one in six of all who contracted it in a "natural way". Nor was the fear of death all; there was also the dread of disfigurement.

Jenner's early life was interesting. At the age of twenty-one, after serving his apprenticeship to a surgeon at Sodbury, near Bristol, young Jenner went to London and lived for two years as a house pupil of John Hunter. This was the beginning of a lifelong friendship between these two. Jenner learned from Hunter's maxim "Don't think, but try; be patient, be accurate."

During his apprenticeship, a young dairymaid had said to him, "Oh! I shall never have smallpox, for I've had cowpox." When Jenner settled in Berkeley in Gloucestershire, he soon found that all the country folk held firmly to this belief whilst all the local doctors laughed at it as an old wives' tale, but Jenner was patient. For twenty-five years, he studied the affections of cow's teats and udders. He found true cowpox rare as compared with the many other conditions simulating it, which were sometimes conveyed from the cows to the hands of the milkers. These spurious diseases did not protect but true cowpox, once sustained, did. At length, the time came for an experimental trial. On 14th May 1796, this undistinguished country doctor successfully inoculated a healthy lad of eight years with cowpox, using secretions from the lesions on the hands of a milkmaid. Sarah Nelmes was the milkmaid and James Phipps the small boy. Eight weeks later, Jenner inoculated Phipps with true smallpox. Nothing happened! The cowpox had prevented the boy from contracting the fell disease.

Two years later, Jenner wrote a book of seventy-five pages entitled *An Inquiry into the Cause and Effects of* Variolae Vaccinae, *a Disease discovered in some of the Western Counties of England, particularly Gloucestershire, and known by the name of Cowpox.* Encouraged by students he had met at John Hunter's, Jenner went to London. At first there was opposition but gradually more and more physicians and surgeons tried. The idea was gradually accepted in England, then in Europe and finally America. He was honoured by Spain, Italy and Denmark, and the Empress of Russia presented him with a diamond ring. At the outbreak of war between Britain and France many Englishmen living in France were held prisoner by Napoleon. All the usual diplomatic requests failed to secure their release. Jenner then wrote direct to Napoleon and the Emperor immediately ordered the release of all prisoners, saying "We can refuse nothing to that man." Fame and reputation were his, but great patients and large fees never came his way. Vaccination was a simple procedure which anyone could perform. Jenner went back to his country practice and worked quietly on till in his seventy-third year he died of apoplexy.

### WILLIAM GILBERT GRACE

Grace was born at Downend, near Bristol, in 1848 and by common consent was the greatest cricketer the game has ever known. During his career of nearly forty years, he twelve times headed the list of batting averages and compiled over 200 "centuries". As a bowler, he was only slightly less successful and when over fifty years of age he still retained a commanding position in first-class cricket. His first great match was in

1864 when he played for South Wales at Kennington Oval, and immediately afterwards he made 170 at Brighton against the Gentlemen of Sussex. In 1868, he scored 100 twice in one match—an achievement that he repeated on three occasions. Engaged in first-class cricket for thirty-six years, he fairly earned the title of Champion. His highest score of 400 not out was made against Twenty-two of Grimsby in 1876. His highest score in first-class cricket was 344. In 1875, he took 192 wickets in one season. Playing against Oxford in 1886, he took all the wickets in one innings for 49 runs. In 1899, he became secretary and general manager of the London County Cricket Club at Crystal Palace. He was largely responsible for establishing cricket as a national pastime as we now know it.

Medical men are always proud to claim him as a member of the profession and I have two original stories to tell you, which I think have not been told before. Both show his sterling human qualities and his skill as a doctor. Dr. Grace was very friendly with another cricketing character, Sammy Woods, and these two often trained together for great occasions. Part of their training consisted in long walks over the Mendip Hills. After such an expedition one very hot summer's day, W. G. became very thirsty and suddenly exclaimed to his companion "What wouldn't I give for a drink of beer, Sammy!" Without answering, his companion disappeared over a stone wall and in a few minutes returned pulling from his pockets two bottles of beer. Grace gazed in amazement at this apparently magical feat. He could only stammer "Where in the world did you get those?" To which Sammy gaily replied, "Oh! that's easy. I've got 'em planted all over these Mendips."

In 1937, at the end of a meeting of a women's guild at which I had been speaking, a very old lady came to me, very proud to tell her story of Dr. W. G. Grace.

It seems that as a young woman she had lived in a very poor part of the city, near Quaker's Friars. Her own doctor, who charged the extraordinary fee of five shillings for a confinement, was apparently very good but rather addicted to the bottle. So much so, that when her time came and her husband went to get the doctor, the latter's wife could only say, "I'm sorry, Mr. So-and-So, but my husband is in no fit state to come." The man, taken aback, was quickly comforted when he was told to go and find Dr. Grace. This meant a considerable journey, late at night but away the man went. Although W. G. was already in bed, he quickly got up and dressed, got out his "dog cart" and with the anxious husband up beside him, drove to the patient's house.

My old lady was apparently better tended than she had ever been before. However, the point that remained so clearly in her mind was that as soon as Dr. Grace saw her husband he said, "Have you any eggs and brandy in the house? She'll want them tomorrow." The man could only dejectedly shake his head. He had none and also lacked the means to get either. But the generous-hearted W. G. was equal to the occasion. Patting the husband on the shoulder, he said, "You come round to my house in the morning and I'll give you some eggs and half a bottle of brandy."

A truly great cricketer, a kindly doctor and a gallant gentleman.

### ERNEST WILLIAM HEY GROVES, 1872–1944

Almost eleven years ago—on 22nd October 1944—Ernest Hey Groves died in Clifton. It is with some diffidence that I mention one who was so recently with us but I do so because I was his surgical dresser and have a vivid recollection of his sterling qualities, both as a surgeon and as a man. Finally, it fell to my lot to assist in caring for him during his long and distressing last illness, during a great part of which he was nursed in Southmead Hospital.

Groves was born in India in 1872 and was the son of a Civil Engineer who settled in Bristol on his retirement, when the boy was only three years old. He went to school in Bristol, gained an entrance scholarship at Bart's, took B.Sc. in London in 1890 and qualified M.R.C.S., L.R.C.P. in 1895. He took his M.B. in 1897 and M.D. in 1900. After being Resident Accoucheur at Bart's under Sir Francis Champneys, he was in general practice in Chewton Mendip. He once said to me "Many is the time I've wandered over those Mendips searching for a hypothetical half-crown." Later, he settled in Kingswood, where he carried out many surgical operations in his own home, and where his wife, who had been a sister in St. Bartholomew's Hospital, helped by nursing the patients. In 1905, he gained high academic honours by taking the London M.S. with Gold Medal and the F.R.C.S., a truly remarkable record for a man engaged in practice.

In the war which came soon afterwards, he went to Egypt in charge of the Surgical Division of a General Hospital and there elaborated his extension splints for fractures of the lower limb and became one of the pioneers in orthopaedic surgery. He was most friendly with Sir Robert Jones who visited him many times in Bristol, thus cementing their common interest in bone surgery.

Perhaps Hey Groves's greatest triumph was the production of the *British Journal of Surgery* and of which he was Editorial Secretary for more than twenty-five years. The *Journal* was founded in 1913, and it was largely through his enthusiasm and industry that it has attained a high position in the world as a surgical publication.

In the very early twenties he, together with the late Dr. Carey Coombs, did his utmost to promote harmony and co-operation between the General Hospital and the Royal Infirmary in Bristol. Amalgamation was much in the air in those days, but the plan misfired and was delayed for over twenty years.

As a surgeon, no one could be bolder when the occasion demanded. The most dramatic operation I can recall was about 1921, when tumours of the lung were rarer than they are today. A Swedish sailor had been found to have such a tumour in the left chest. Of course, the question of aneurysm had been considered but this seemed too big for such a diagnosis, and finally physicians agreed that biopsy was the only answer. At least fifty persons crowded into the theatre to see this unusual sight. The tumour was displayed by a wide incision and a trap door of ribs raised and a section taken, the report being returned by the pathologist a few days later as "laminated clot"! However, the patient survived for several months before dying of a sudden haemoptysis.

From 1922–32, Hey Groves was Professor of Surgery and his work was recognized when the University conferred upon him an Hon. D.Sc. at the Centenary of the Bristol Medical School in 1933.

His *Synopsis of Surgery* is well known and he published many other books for students and nurses besides being a prolific writer in the journals.

He was a good billiard player and a fine swimmer, especially under water. He could dive in off the highest board at Henleaze Lake and amaze everyone by the length of time that elapsed before he "surfaced" again. As a host and companion, he was unsurpassed. He loved the novelty of travelling and visited Australia for the B.M.A. meeting in 1935, being President of the Orthopaedic Section on that occasion.

The final phase of his work came when he was group officer for Bristol in the E.M.S. at the outbreak of the Second World War. All his old enthusiasm was revived at this time and it was a shock to his many friends and colleagues when he collapsed from a stroke which rendered him helpless for so many months. So passed a great English surgeon whose name is inseparably connected with Bristol and the West.

